# Risk Factors and Biomarkers for Chronic Hepatitis B Associated Hepatocellular Carcinoma

**DOI:** 10.3390/ijms22020479

**Published:** 2021-01-06

**Authors:** Vijay Pandyarajan, Rajalakshmi Govalan, Ju Dong Yang

**Affiliations:** Division of Gastroenterology and Hepatology, Samuel Oschin Comprehensive Cancer Institute, Comprehensive Transplant Center, Cedars-Sinai Medical Center, Los Angeles, CA 90048, USA; Vijay.Pandyarajan@cshs.org (V.P.); Rajalakshmi.Govalan@cshs.org (R.G.)

**Keywords:** HBV, HCC, liver cancer, risk factor, biomarker, early detection, liquid biopsy

## Abstract

Globally, hepatitis B virus (HBV) related hepatocellular carcinoma (HCC) is one of the major causes of cancer-related mortality. This is, in part, due to delayed diagnosis and limited therapeutic options with more advanced stages of the disease. Given the prognostic importance of early diagnosis, novel methods for early detection are in need. Unlike most other cancer types, tissue is not required to diagnose HCC and is frequently avoided given the inherent risks of liver biopsy, so less invasive methods of obtaining tumor material are currently under investigation. Material shed from tumors into the periphery are being investigated for their potential to both surveil and diagnose patients for HCC. These materials include circulating tumor cells, DNA, RNA, and exosomes, and are collectively termed a “liquid biopsy”. In this review article, we discuss the evolving literature regarding the different risk factors for HCC and the types of emerging novel biomarkers that show promise in the prevention and early diagnosis of HCC within the context of HBV infection.

## 1. Introduction

Globally, hepatitis B virus (HBV) is a leading cause of chronic liver injury, leading to cirrhosis [1]. HBV has the highest prevalence in parts of Asia and Africa, where it is frequently found to be vertically transmitted from mother to child. Immune response directed towards the virus is the primary cause of hepatic injury [2]. Given its high oncogenic potential, screening for hepatocellular carcinoma (HCC) in those with chronic HBV infection are recommended in select populations, particularly those of Asian or African descent (Table 1) [3]. As there is currently no cure for HBV infection, care has been directed mainly at prevention and, if infected, suppression of the virus.

HCC continues to be one of the leading causes of cancer-related deaths throughout the world, and frequently presents itself within the context of cirrhosis [5]. This has also led to the development of guidelines among all major liver societies for HCC surveillance, given its potential for cure in earlier stages [4,6]. As HCC is often found at advanced stages, there has been a strong drive to develop modalities to detect HCC at an early curable stage. As a diagnosis of HCC can be made radiologically, biopsy is often not required. Newer techniques to both detect and classify HCC are the subject of investigation and are of urgent need. This article aims to review the latest literature regarding risk factors, risk stratification, and non-invasive diagnostic methods for HBV-related HCC, with an emphasis on novel biomarkers and liquid biopsy.

## 2. Risk Factors and Risk Stratification for HCC

### 2.1. Host factors

#### 2.1.1. Age

Older age is a strong risk factor for the development of HCC. The incidence of HCC increases with age. For example, patients in the age range of 30 to 39 only had an HCC incidence rate of 197/100,000 person-years, whereas it was 927 for those in the 60–69 year-old age group [7]. This finding has been confirmed in more recent studies of 820 chronic HBV patients who were followed for a mean duration of 76.8 months [8,9]

#### 2.1.2. Sex

The prevalence of HCC is higher in men than in women. In a large study involving 854,000 HCC incident cases worldwide in 2015, around 591,000 cases were men. [10] At a global level, this ratio was found to be the highest in East Asia. Given that male sex has been established as a risk factor for HCC, it has been incorporated into the cirrhosis, age, male sex, and diabetes (CAMD) score, which is used to predict the risk of the development of HCC during treatment with antiviral therapy [11,12]. Women may be protected from the development of HCC through estrogen-mediated effects, while androgens may promote oncogenesis in men [13,14,15,16]. The gender disparity is of great interest and is an area of active research.

#### 2.1.3. Genetics/Ethnicity

Host genetic factors play a critical role in the development of HCC in patients with HBV. Cohort studies have examined the role of a family history of HCC and found that patients with first-degree relatives with HBV-related HCC were at a higher risk of developing HCC [17,18,19]. Ethnic background is also an independent predictor of HCC in the context of HBV-related HCC, which is why the American Association of the Study of Liver Disease (AASLD) recommends early screening for patients of Asian or African American descent (Table 1) [4]. This may be due to the greater prevalence of the vertically transmitted virus in these populations, which leads to a longer duration of infection. Polymorphisms within the *IL16* gene have been identified within a group of Chinese patients, which is associated with HBV-related HCC [20]. The mechanisms underlying genetic host and viral factors is an area of ongoing research.

#### 2.1.4. Metabolic Syndrome

Metabolic syndrome is a constellation of signs and symptoms, which include obesity, hypertension, hypertriglyceridemia, and the presence of diabetes. The rising incidence of these conditions worldwide has been paralleling the increasing access to Westernized-type diets, high in carbohydrates and fats. The correlation between HBV and obesity/diabetes and HBV-HCC was examined in a large patient cohort based in Taiwan [21]. Obesity by itself did not correlate with the development of HCC in those with HBV infection, although diabetes was found to confer a 2–3 fold increased risk for the development of HCC over those without diabetes. Interestingly, a combination of both obesity and diabetes increased the relative risk of development of HCC to 264.7 in those who were HBsAg positive. This markedly increased risk is thought to be due to the synergistic effect of obesity/diabetes and HBV, which can lead to a state of increased inflammation and oxidative stress, and subsequently to the development of non-alcoholic fatty liver disease (NAFLD). HBV in the setting of NAFLD can accelerate the development of HCC. Similar results were also seen within a cohort of men from the Risk Evaluation of Viral Load Elevation and associated liver disease/cancer in HBV (REVEAL-HBV) study, which showed that, although obesity by itself did not increase the risk of HCC, obesity and simultaneous alcohol use increased the relative risk to 3.1 [22]. Only one study by Tung et al. was unable to find an association between diabetes or obesity with the development of HCC, although this was done in a dual HBV/HCV infected population [23].

### 2.2. Environmental Factors

#### 2.2.1. Smoking

DNA damage caused by chemicals found in tobacco smoke is the primary driver for the development of cancer [24]. There appears to be a synergistic effect of tobacco use with chronic HBV infection, leading to a greater risk for the development of HCC. In a meta-analysis of 16 studies performed in 2010, the risk of HCC increased from 15.8 to 21.6% for HBV positive individuals who smoked [25]. Furthermore, smoking was associated with HCC risk in a dose-dependent manner among those infected with HBV [26]. The authors of this study found that the number of pack-years of smoking correlated with a higher viral load, which they posit increases the risk of HCC development.

#### 2.2.2. Aflatoxin

Aflatoxin is a well-established hepatocarcinogen produced by species within thegenus Aspergillus, which is frequently found in uncooked food. The main pathophysiological mechanism by which aflatoxin is thought to induce cancer is through the formation of specific DNA adducts driven through a DNA conformational specific process [27]. A mutation in the *TP53* gene where a serine residue is substituted for the naturally occurring arginine has, for example, been found in sub-Saharan African and South-Eastern Asian populations, where HCC is in much higher prevalence and is thought to be due to the direct mutagenic effect of aflatoxin. This mutation was found to be in a higher proportion of those with HBV in a population study carried out in Gambia [28]. Several proposed mechanisms have been suggested that potentially show an interaction between HBV and aflatoxin, including the altered metabolism of aflatoxin and the induction of chronic inflammation, which increases cell turnover and enhances the risk of obtaining an oncogenic mutation [29].

Aflatoxin or its metabolites are detectable in both serum or urine, and large studies have shown a correlation between the incidence of HCC and aflatoxin. Urinary aflatoxin biomarkers were detected in 50 out of the 55 patients who developed HCC over 3 years in a study involving a cohort of 18,244 men [30,31]. In a more recent case-control study, chronic HBV patients were monitored for over 20 years, and a significant dose–response relationship was described between the quantity of measured serum aflatoxin-adducts and the development of cirrhosis or HCC within 9 years of study entry [32]. Several HBV related risk factors were identified in this study. In a stratified analysis, an increased risk of HCC was seen in patients with HBeAg seropositivity, HBsAg level >1000 IU/mL, or HBV DNA copies/mL >10,000. Aflatoxin has, therefore, become an important environmental factor that can lead to the development of HCC in the HBV population. In clinical practice, aflatoxin or its metabolites are not frequently measured, as there are no therapies to reverse the DNA damage that has already occurred.

#### 2.2.3. Aristolochic Acid

Aristolochic acid is a mutagen and nephrotoxin present in the *Aristolochia* and *Asarum* plant genera, which are frequently used as herbal medicines. This potent toxin primarily induces adenine to thymine mutations [33]. An interaction between aristolochic acid and HBV was suggested in a study of 802,642 patients with HBV infection, which examined aristolochic acid exposure and HCC incidence. A strong dose-dependency was found between the amount of aristolochic acid consumption and the development of HCC within this cohort [34]. A significant increase in the adjusted hazard ratio (1.61) was found in those who had used more than 1000 mg of aristolochic acid. Because of the strong association with not only HCC, but bladder cancers, aristolochic acid containing herbal medications have been banned in many countries.

### 2.3. Viral Factors

HBV promotes HCC through both direct and indirect mechanisms. One of the primary mechanisms by which cancers can develop is through repeated cycles of cell death and regeneration, and the initiation of an inflammatory cascade, which can allow for the propagation of oncogenic mutations [35]. At the DNA level, the HBV genome can integrate itself into host genes that can provide a growth advantage to the host cell. HBV has been found to be integrated into or near cancer-related genes, such as *TERT*, *MLL4,* and *CCNE1,* which have all been shown to be upregulated in tumors [36]. HBV has also been found integrated into numerous other genes that can promote HCC [37].

The translated protein products of the viral HBV genome have also been implicated in promoting carcinogenesis. The HBV circular DNA encodes several proteins, including the surface antigen (HBsAg); core antigen (HBcAg), which is the main component of the nucleocapsid; and HBeAg, which is a splice variant of the core protein, a DNA polymerase and HBx protein a regulatory protein required for viral replication. Each of these proteins or their precursors have been shown to promote cancer through alteration of the signaling pathways that govern the cell cycle within hepatocytes [38].

HBx, for example, has been extensively studied and has been shown to interact extensively with host molecules. Experiments done with HBx-deficient hepatitis B particles did not lead to any production of HBV, indicating how critical this protein is for viral replication [39]. Various mechanisms have been proposed to explain how HBx influences viral replication and oncogenesis. These include, and are not limited to, acceleration through cell cycle checkpoints to increase cell proliferation, activation of Ras pathways, and direct stimulation of viral replication [40,41,42]. Although direct links between HCC and HBx function have not been found, the aforementioned mechanisms may indirectly influence the development of HCC [43]. One mechanism in particular that has been well elucidated includes the binding of HBx to damage-specific DNA-binding protein (DDB1), which includes an E3 ubiquitin ligase that is then redirected towards the degradation of SMC5/6, a transcriptional repressor of the HBV covalently closed circular DNA (cccDNA) [44]. The HBx protein has also been found to directly interact with the B-cell lymphoma 2 (Bcl-2) and the B-cell lymphoma-extra-large (Bcl-xL) proteins, which subsequently increases intracellular calcium and greatly enhances viral replication. Additionally, this interaction increases the rate of cell death [45]. This interaction is through a B-cell homology domain-3 like (BH3) structural motif found on HBx, whose structure was recently more clarified [46].

The ability of virally translated products to influence the development of HCC has been investigated in large cohorts of patients. Hepatitis B e antigen is a protein of unclear function that is found in measurable quantities in the periphery during active viral replication. The development of antibodies against HBeAg indicates a decline in viral replication. The role in determining the presence of HBeAg and its association with HCC was best described in a large prospective study including 11,893 men in Taiwan; 39% of those who tested positive for HBeAg and HBsAg were found to have developed HCC [47]. HBV DNA levels greater than 10,000 copies/mL was itself also found to be an independent predictor of the development of HCC. [48] A model based on the viral profile, as well as patient characteristics, was developed from the data acquired from the REVEAL-HBV cohort in order to determine the 5-,10-, and 15- year risk of HCC with area under the receiver operating characteristics (AUROCs) within the 0.84 to 0.89 range [49].

Hepatitis B has at least 10 known genotypes (A-J), which are prevalent at different frequencies throughout the world and are correlated to clinical outcomes within these patients [50,51]. Of particular relevance to HCC, genotype C has been shown to have the highest risk for the development of cancer [52]. This is thought to be mainly as a result of this strain’s particular ability to replicate more easily than other genotypes [53]. Knowledge of the exact strain of HBV plays no role at present in determining treatment regimens, and thus is not routinely tested in clinical practice.

### 2.4. Risk Stratification Models

Ideally, one would like to identify patients at high risk of developing HCC (see Table 2) based on clinical parameters in order to more aggressively treat or monitor these individuals. To that end, several risk prediction models have been developed.

Using data from the REVEAL-HBV cohort, a 17-point risk score was developed to predict HCC occurrence. Termed the REACH-B score, this model had an AUROC that ranged from 0.796 to 0.902, depending on the cirrhosis status of the patient [54]. This was validated in a cohort of 1505 patients from Hong Kong and South Korea, and used the variables of sex, age, alanine aminotransferase (ALT), HBeAg status, and HBV DNA to produce their risk model.

Another HCC risk model was also derived from the REVEAL-HBV cohort [49]. This model used similar clinical parameters to the previously mentioned REACH-B risk score, but also incorporated the HBsAg load and genotype into its calculations. The AUROC for predicting the 5-, 10-, or 15-year risk for development of HCC ranged within 0.84 to 0.87.

Two other risk calculators, one with age, gender, HBV DNA, core promoter mutations, and cirrhosis (GAG-HCC) and the Chinese University HCC score (CU-HCC) were also developed to predict HCC [8,55]. As in the previous REVEAL-HBV study, these scores were derived from Asian populations. The AUROC for the GAG-HCC risk calculator had a range of 0.88–0.89, while a cutoff value of 5 for the CU-HCC risk score had a negative predictive value of 97.3%. Both risk calculators have several factors in common with the previously mentioned studies.

Given that the populations for the previously mentioned studies comprised of those of Asian descent, there was some concern that these risk models may not have broader applicability to other populations. In fact, when these models were applied to Caucasian patients with chronic HBV infection, there was poor predictability for the development of HCC [56,57]. To that end, a separate risk score was developed for the Caucasian population. In a population of 1815 Caucasian adults with chronic hepatitis B and no HCC, who received treatment with entecavir or tenofovir for more than 12 months, an HCC risk prediction score was developed based on age, gender, HBeAg status, body mass index (BMI), ALT, platelet count, HBV viral load, prior treatment with interferon or nucleos(t)ide analogues other than entecavir/tenofovir, and the presence of cirrhosis. Termed the PAGE-B risk score, this initial multivariate model was then simplified to an integer scoring system that only included, age, gender, and platelet count. Patients with a risk score greater than 18 were found to have a cumulative five-year HCC incidence rate of 16%, as seen in the validation dataset.

## 3. Serum Protein Biomarkers for HCC

After several decades of chronic HBV infection, about 20–30% of patients with cirrhosis develop HCC [58]. There is a significant proportion of HBV infected patients without cirrhosis who end up developing HCC [59]. In both cases, a larger tumor burden is associated with a poor prognosis. Hence, early diagnosis helps to detect the tumors at a curative stage with improved chances of successful treatment and prolonged survival [58,60]. The current European Association for the Study of the Liver (EASL) suggests using abdominal ultrasound (US), while the American Association of the Study of Liver Disease (AASLD) recommends US with or without alpha-fetoprotein (AFP) as a surveillance modality for HCC at six-month intervals [4,6]. However, about 20% of ultrasound studies are classified as inadequate for surveillance, and only have a sensitivity of 63% for early HCC when combined with AFP. They also have high intra- and inter-operator variability [4,61]. We are therefore in need of reproducible, less invasive biomarkers to help detect the presence and progression of HCC [58].

### 3.1. Alpha-Fetoprotein (AFP)

AFP is the most widely used traditional protein biomarker. It is a 70 kDa glycoprotein produced by the fetal yolk sac and liver [62]. The serum levels of AFP are high at the time of pregnancy and in newborns, but quickly decrease after birth. Pathologic elevation of AFP in adult life is seen with acute hepatitis, endometrial sinus tumors, and HCC [63]. The level of AFP elevation correlates with HCC tumor size, portal vein tumor invasion, treatment response, and post-transplant recurrence of HCC, making it an important prognostic and predictive biomarker [64]. However, its role in the early detection of HCC is limited by its poor sensitivity of less than 60% when a threshold of >20 ng/mL is used [4], highlighting the urgent need to develop a better diagnostic biomarker for the early detection of HCC.

### 3.2. Lens Culinaris Lectin-Binding Sub-Fraction of the AFP

There are three different glycoforms of AFP, AFP-L1, AFP-L2, and AFP-L3, each with an increasing binding affinity to *Lens culinaris* agglutinin. AFP-L3, with the highest binding affinity, is secreted by malignant HCC cells in the early tumor stages, even in the absence of elevated AFP, making it a promising early screening tool [61,65]. The fraction of AFP-L3 to total AFP, termed as AFP-L3%, correlates with the degree of malignancy and has been associated with aggressive forms of HCC [66,67]. Leerapun et al. demonstrated that AFP-L3% increased the specificity of HCC diagnosis to 100% in those patients with an indeterminate AFP value (10–200 ng/mL) and an AFP-L3% greater than 35% [67]. While multiple studies have evaluated the clinical utility of AFP-L3% as a potential screening and diagnostic tool, they have used varying cut-offs, test methods, and patient populations, yielding variable sensitivities ranging from 21–84% and specificities from 89–94% [61]. Thus, larger cohorts with consistent methodologies are needed to reliably demonstrate the superiority of AFP-L3% over AFP.

### 3.3. Des-Gamma Carboxy Prothrombin (DCP)/Protein Induced by Vitamin K Absence (PIVKA-II)

Prothrombin is one of the coagulation factors synthesized by the liver. It is dependent on vitamin K for carboxylation at glutamic acid residues, which is necessary for normal function. When there is a defect in the carboxylation system (e.g., absence of vitamin K, presence of vitamin K antagonists like warfarin the non-carboxylated form is released [68]. This non-carboxylated form of prothrombin is known as des-gamma carboxy prothrombin (DCP), which is also referred to as the protein induced by vitamin K absence (PIVKA-II) [69]. Elevated levels of DCP produced by the HCC cells were thought to result from an acquired post-translational defect in hepatic vitamin K dependent carboxylation [69].

It was first identified in 1984, primarily as a serum marker in association with HCC. In more recent years, a role for the promotion of HCC itself is now being identified [68]. It is unclear at present what specific mechanisms increase the production of DCP, but hypoxia may be one such factor, a condition frequently found within the cores of tumors. This has been examined in cell-based studies that revealed increased production of DCP under hypoxic conditions [70,71]. DCP may also serve as a paracrine hormone to induce the growth of endothelial cells necessary for angiogenesis, as well as a mitogen to stimulate the growth of HCC cells itself [72,73].

Given the known biology of DCP, it has become of great clinical interest because of its potential diagnostic and prognostic value. In terms of diagnosis, the sensitivity and specificity for HCC detection with DCP has been reported to be 75.1% and 94.8%, respectively, with a cut off of 40 mAU/mL. DCP has also been studied extensively in terms of its prognostic value. HCC with higher DCP levels is indicative of worse clinical features. [74,75]. Jun Ji et al. have also demonstrated that DCP can be used in identifying AFP-negative HCC, and has improved performance in HCC surveillance, early diagnosis, treatment response, and recurrence monitoring in the HBV-related population [76]. Patients with high levels of DCP/PIVKA-II prior to treatment were found to have higher tumor recurrence and lower disease-free survival post curative-treatment within the context of HBV-HCC. When definitive curative therapy is not an option, particularly in patients with advanced HCC, sorafenib, a tyrosine-kinase inhibitor, may be employed as a first line of therapy [77]. PIVKA-II levels post-treatment with sorafenib have shown mixed results, either not decreasing or even increasing soon after treatment [78,79] One study including 50 patients with HCC treated with sorafenib showed that an increase in DCP greater than 2-fold from pre-treatment levels had a longer time to progression, which was significantly longer than those without an increase in DCP [80]. The authors posit that the inhibition of angiogenesis by sorafenib leads to hypoxia within the tumor, which then induces production in DCP. Cases of complete remission of HBV-HCC treated with sorafenib, on the other hand, have also shown a complete normalization of DCP [81]. What can be surmised from these studies is that an initial increase in DCP soon after treatment, with the eventual complete normalization of DCP, likely portends the best prognosis.

### 3.4. Combination of AFP, AFP-L3, and DCP

Multiple studies have evaluated and compared the performance of the aforementioned biomarkers (*AFP*, *AFP-L3*, and *DCP*), the most commonly used serum biomarkers for HCC detection. This has been examined in 240 patients with HBV or HCV, with or without HCC [82]. Serum levels of DCP, AFP, and AFP-L3 were all found to be significantly higher in those patients with HCC than those without. Values of 84 mAU/mL; 25 ng/mL; and 10% for DCP, AFP, and AFP-L3 were found to have the best ability to identify those with HCC. Of the three values, DCP was found to be normal in patients without HCC and had the best ability to distinguish those patients with HCC. The use of DCP alone had a sensitivity of 87% and specificity of 85%. Additionally, DCP had a better correlation with the size of the lesion. A similar finding for the utility of DCP was also evaluated by Yoon et al., which found a 51.9% sensitivity of DCP with a specificity of 97%. A combination of AFP and DCP resulted in a sensitivity of 78.3% [83].

This was more recently evaluated in a large phase 3 study, which sought to assess the performance of AFP, AFP-L3, and DCP for the detection of HCC in the earliest stages [84]. In this study, 689 patients with cirrhosis and/or chronic hepatitis B were followed for a median of 24.1 months. Of those, 42 patients developed HCC and were matched to those who did not have HCC. The best combination of biomarkers that differentiated the cases from the controls at the time of diagnosis was a combination of AFP, AFP-L3, and DCP with an AUROC of 0.86. The novel findings from this study are the inclusion of biomarkers at 6 and 12 months prior to the diagnosis of HCC. Although not as robust, the highest AUROC of 0.78 and 0.71 was shown for a combination of AFP and AFP-L3 at 6 and 12 months, respectively. The authors found that AFP and AFP-L3 began to rise approximately 6 months prior to the diagnosis of HCC, and that these values remained the same in those who did not have HCC.

### 3.5. Golgi Protein Complex 73 (GPC 73)

Golgi protein complex 73 (GPC 73) is a 73 kDa transmembrane glycoprotein found in the cis-Golgi complex. It is expressed in a number of epithelial cells, including normal biliary epithelial cells, but not by normal hepatocytes [85]. In cases of liver diseases, including hepatitis B, cirrhosis, and HCC, the hepatocytes, but not the biliary cells, undergo transformation to upregulate the expression of GPC 73 [86]. Studies have used immunohistochemistry, Western blot, and Enzyme-Linked Immunosorbent Assay (ELISA)as detection methods with comparable efficacies [87,88,89]. In a meta-analysis performed by Dai et al., the diagnostic accuracy of GPC 73 was superior to AFP, and the combination of GPC73 and AFP had a higher accuracy than either biomarker alone, suggesting its role as a diagnostic and prognostic tool in HCC [89]. Xu et al. demonstrated a positive correlation between the serum GPC 73 levels and liver disease severity in chronic hepatitis B patients. The sensitivity and specificity of GPC 73 for HCC detection were 79% and 80%, respectively, at a cut-off of 138.3 ng/mL [88]. GPC 73 was also found to be a useful marker for the detection of HCC in a population of HBV patients with an AUROC of 0.89, compared with AFP within this study (0.77). Additionally, RT-PCR of the GPC73 gene was found to be superior for the direct detection of GPC73 with an AUROC of 0.92 [90].

### 3.6. Osteopontin (OPN)

Osteopontin (OPN) is an extracellular matrix integrin-binding glycoprotein involved in immune, tumorigenic, and bone homeostasis. They are normally expressed in bones, teeth, and epithelial biliary cells, but are absent in normal hepatocytes. The expression of OPN is increased in HCC, but not in cirrhotic liver or viral hepatitis [91,92,93]. In a meta-analysis by Shang et al. at a threshold of 91 ng/mL, OPN had a sensitivity of 74% for the detection of HCC compared with 53% for AFP. The authors concluded that the performance of OPN is better than AFP in terms of sensitivity. When combined with AFP, their sensitivity and specificity increased to 95% and 96%, respectively [92]. Wan et al. performed a meta-analysis that showed that OPN has comparable accuracy to AFP in HCC detection [94].

In a study by Xie et al., over-expression of OPN was found in 39 of 72 patients who underwent hepatectomy for HBV-related HCC [95]. High expression levels as determined by immunohistochemical staining were found to correlate with capsular and portal invasion, as well as lymph node metastasis. Furthermore, patients with tumors that highly expressed OPN had a disease-free survival of only 14 months, compared with 36 months for those without. Overall survival also correlated with OPN expression. Those with a high expression of OPN had a survival of 18.6 months compared with 42.6 months.

## 4. Novel Blood-Based Biomarkers for HCC

While sampling an HCC tissue is typically not required for diagnosis given its inherent risks, little can be gleaned about tumor biology without primary tissue. Newer methods termed “liquid biopsies” have been developed to, detect malignant tissue or products from body fluids such as peripheral blood without the need for a direct biopsy. These techniques involve direct detection of circulating tumor cells (CTC), circulating tumor or cell-free DNA (ctDNA/cfDNA), tumor-associated microRNAs, or extracellular vesicles (EV) produced by the tumor.

### 4.1. Circulating Tumor Cells (CTCs)

CTCs are cells that have been shed by the primary cancer into the peripheral circulation. The detection of these cells is difficult, given the low frequency of these cells when compared with the plethora of non-cancerous host cells (i.e., blood cells). Current detection methods include binding to cell surface receptors, such as the epithelial cell adhesion molecule (EpCAM), which is only found on epithelial and not hematopoietic cells [96]. Currently, the only FDA-approved test for detecting CTCs, the Cell-Search system™, utilizes this cell surface marker. Unfortunately, the number of cells detected in the context of HCC is low and so it is not currently integrated into clinical practice. Newer methods that utilize microfluidics or other cell surface markers are all under current investigation. [96]

The use of CTCs was investigated in the context of HBV-related HCC in a small patient population of 42 individuals in China with HBV-related HCC who underwent surgery [97]. Preoperatively, no correlation was made with the number of CTC to most of the clinical parameters that were tested, except for Edmondson stage. However, those with more than two or five CTCs per 7.5 mL were found to have a significantly higher risk of recurrence, with a hazard ratio of 8.72 and 6.89, respectively. Much work still needs to be undertaken to understand how CTCs can be used to understand HBV-induced HCC.

### 4.2. Cell-Free/Circulating Tumor DNA (cfDNA/ctDNA)

Cell-free DNA (cfDNA), as the name implies, are cell-free nucleic acids found in peripheral blood. When produced by tumor cells, the term circulating tumor DNA (ctDNA) is used. Techniques for analysis include digital droplet PCR, which can provide more accurate quantitation or next generation sequencing, which can target a whole genome. Several techniques, such as specific PCR primers or next-generation sequencing, have been utilized to amplify and detect low-frequency sequences. It has been shown, with HCC, that the amount of peripherally found tumor DNA reflects the aggressiveness of the tumor [48,98]. Both of these techniques have been utilized in the detection of HCC. Epigenetic changes of ctDNA have also been used as a method to detect HCC. These include the methylation of *SEPT9*, which was originally designed to detect colon cancer, and so is non-specific [99].

Specific data regarding the use of liquid biopsy techniques in HBV patient populations have been limited. One study by Qu et al. examined a population of 3793 HBV infected individuals using a liquid biopsy technique [100]. Patients were initially screened using traditional methods and then had a screening procedure that utilized a combination of serum markers and cell-free DNA. The serum markers included AFP and DCP. Specific genetic sequences found in HBV-associated HCC DNA were used as probes. This test was able to discriminate early-stage HCC from non-HCC patients, with a sensitivity of 85% and a specificity of 93%. When this algorithm was tested against individuals who initially tested negative for HCC with imaging studies, a positive predictive value of 17% was calculated, with a sensitivity of 100% and specificity of 94%. Furthermore, when the tumor size was evaluated in the HBV positive patients, the sizes of these lesions were within 2–3 cm. The authors concluded that this liquid biopsy technique could be used to detect and diagnose early-stage HCC.

The quantity of circulating tumor DNA in the context of HBV was evaluated by Chen et al. [98]. CtDNA was obtained in a patient population of 210 individuals, of whom 160 had chronic HBV infection. Patients with HCC were then compared to those without HCC. Significant differences were found in the concentration of cfDNA between healthy controls and those with HCC. However, this finding did not extend to patients with hepatitis B without HCC compared with those with HCC, as the AUC was only 0.56. The authors posit that the negative result could be due to a high level of inflammation and cell death in the setting of chronic HBV infection. What was found to be significant in discriminating HBV patients with HCC from those without was the integrity of DNA with an AUC of 0.83. The authors postulated that cfDNA integrity may be a better marker for the detection of HCC.

The correlation between the cfDNA concentration and HBV-related HCC was also examined recently [101]. Concentrations of cfDNA were negatively correlated with serum albumin, but positively correlated with their prothrombin time and tumor diameter. Using multivariate analysis, a correlation was shown between the Childs–Pugh–Turcott class and the total concentration of DNA. Those patients with a higher total plasma cfDNA were found to have a poor prognosis and increased risk of early HCC recurrence.

### 4.3. Tumor-Associated MicroRNAs (miRNA)

While only 2% of the human genome codes for mRNA and is translated into functioning proteins, the majority of human genome is composed of non-coding sequences that regulate gene translation. MicroRNAs (miRNA) are non-coding RNA molecules that can range from 19 to 25 nucleotides [102]. Circulating miRNA has remarkable stability (resistance to temperature, pH, and RNases) which make isolation from the peripheral blood less cumbersome using standard RNA purification techniques [103]. Normally in the nucleus of cells, DNA is first transcribed by RNA polymerase II into primary miRNA, which will then be cleaved by a microprocessor complex to pre-miRNA. This pre-miRNA is then exported into the cytoplasm, where it is further processed and unwound to form single-stranded miRNA. It then interacts with other proteins to form an RNA induced silencing complex (RISC), which then acts as a negative regulator of mRNA translation and silences the genes via several proposed mechanisms [104]. MiRNAs are thought to play a significant role in many biologic processes, including metabolism, cell proliferation, cell cycle control, apoptosis, and differentiation. Alterations in the expression of miRNA can therefore result in dysregulation of the cell function [105].

Multiple miRNAs have been associated with different types of malignancy, including colorectal, breast, and cervical cancer, as well as Hodgkin’s lymphoma [105]. Several studies have demonstrated a correlation between serum miRNAs and HCC [106]. In the context of HBV-related HCC, Yu et al. investigated the expression profile of miR-150 in HBV-HCC patients, and found significantly reduced levels when compared with healthy controls and even chronic HBV patients without HCC (82.5% sensitivity and 83.7% specificity in identifying HCC from healthy controls, and 79.1% sensitivity and 76.5% specificity in identifying HCC from HBV) [107]. Moreover, their levels increased after surgical resection and decreased after tumor recurrence. Univariate and multivariate cox regression analysis showed that the serum levels of miRNA are an independent risk factor for overall survival, with lower levels associated with decreased survival. MicroRNA-101 has also been investigated within this context and has also been found to show decreased serum levels in patients with HBV-HCC [108]. Wu et al. evaluated the prognostic impact of the expression and polymorphism of miR-224 in HCC and found that the levels were increased in HCC compared with the healthy cohorts. In the subgroup analysis, the HCC related to HBV had higher levels compared with other etiologies. They also found a polymorphism of miR-224 that was an indicator of liver injury and a prognosticator for survival after liver resection in HBV-related HCC [109].

Another miRNA commonly associated with HCC is miR-122, which makes up a significant proportion of the miRNAs found in the liver [110]. Similar to the previously mentioned studies, miR-122 was found to be downregulated in HBV-HCC patients and was found to be more downregulated in poorly differentiated tumors [111]. The authors found direct evidence that the loss of miR-122 may predispose the tumor to acquire a more invasive phenotype in cell line experiments that was reversed when miR-122 was restored, which may correlate with the poor prognosis of these tumors. MicroRNA-122 was also used in a diagnostic panel of several other microRNAs to detect HBV-related HCC, which showed a significant decrease when subjected to multivariate analysis [112]. When combined, this panel had an area under the curve of 0.89 in the validation dataset. Not all studies have shown a decreased amount of miR-122 within this context. Some studies have shown an increase in mIR-122 in patients in HCC, which may come from damaged hepatocytes [113,114]. A further understanding of the tumor biology may determine if miRNA-122 may serve as a good biomarker for HCC detection.

There have been a number of studies profiling the whole miRNA expression to identify miRNAs correlated with the presence of HCC [106]. In a three-stage study that included healthy controls, inactive HBsAg carriers, chronic hepatitis B, and hepatitis B patients with HCC, qPCR was utilized to identify 19 miRNAs that were increased in HCC patients. The data were then used to build a classifier and were validated in two cohorts of patients and controls. These miRNAs included miR-29a, miR-29c, miR-133a, miR-143, miR-145, miR-192, and miR-505 [115]. This classifier, termed C_mi_, was able to detect HCC with better accuracy than AFP within the validation cohorts, with an AUC of 0.817–0.818. Additionally, this classifier detected HCC well before the clinical diagnosis was made, with a sensitivity of 29.6% at 12 months, 48.1% at 9 months, and 55.6% at 3 months before diagnosis. The authors also demonstrated that C_mi_ was able to identify smaller lesions than AFP could.

Another study to diagnose HCC using miRNAs in chronic hepatitis B patients was also carried out by Jin et al. more recently [116]. Five miRNAs (miR-122-5p, miR-100-5p, miR-125b-5p, miR-885-5p, and miR-148a-3p) were all found to be upregulated in HCC patients compared with the controls. miR- 34a-5p was found to be a potential cirrhotic biomarker, and four miRNAs (miR-1972, miR-193a-5p, miR-214-3p, and miR-365a-3p) were useful for distinguishing HCC from other non-HCC individuals. Furthermore, another six microRNAs were found to be potential prognostic biomarkers, as they were correlated with overall survival and tumor biology. Although miRNA is promising in early detection and prognostication, only a few studies have found a consistent set of miRNAs correlated with early-stage HCC detection. Hence, we need standardized detection methods to improve sensitivity and specificity in order to develop a validated miRNA panel to serve as a tool for diagnosis and prognostication.

### 4.4. Extracellular Vesicles (EVs)

Extracellular vesicles (EVs) are a heterogeneous group of membranous “cargo” vesicles comprised of proteins, lipids, RNA, and miRNA packaged within a lipid bilayer. They are released into the extracellular environment from healthy, inflamed, malignant, or dying apoptotic cells [117,118,119]. EVs are classified based on their biogenesis and size per minimal information for studies of extracellular vesicles (MISEV) 2018 guidelines into small EVs (also called exosomes, with sizes <100 nm or <200 nm) and large EVs (also called microvesicles (MVs), with sizes >200 nm) [120]. Exosomes originate from the fusion of multivesicular endosome (MVEs) with the plasma membrane, while microvesicles are shed directly from the plasma membrane via the membrane budding process [121].

With increasing the understanding of the role of these extracellular vesicles, what was once thought to be a cellular by-product of biological insignificance, is now being explored for its profound clinical utility [122]. EV cargo composition depends on the pathologic and physiologic state of their cells of origin. They are released into the extracellular environment and can be detected in the serum, plasma, urine, saliva, etc. [117]. Moreover, they are protected from degradation by the lipid bilayer, increasing their resistance to RNases, making them an attractive noninvasive liquid biomarker to provide a snapshot of their cells of origin [117,123,124]. Once released into the extracellular environment, they serve as intercellular communication portals via direct receptor activation or indirectly via internalization into target cells. This also makes them a candidate with therapeutic potential [119].

The liver is a multicellular organ made of parenchymal (hepatocytes) and extra-parenchymal cells (Kupffer cells (KC), hepatic stellate cells (HSCs), and sinusoidal endothelial cells (SECs)), each capable of producing different types of EVs, and hence hepatic EVs play a key role in intercellular communication in order to maintain homeostasis [125]. In addition, various hepatic processes, including hepatocyte apoptosis, stellate cell activation, liver innate immune system activation, systemic inflammation, and physiologic stress associated with liver diseases result in the release of different levels of EVs [126]. They serve as biomarkers that aid in the diagnosis and prognosis of several liver diseases including alcoholic and non-alcoholic steatohepatitis (NASH), HBV, HCV, cirrhosis, and HCC [126,127]. The main EV detection methods used in these studies were high sensitivity flow cytometry, ELISA, and qRT-PCR. Thus, methods of isolation, detection, and characterization need to be standardized in the future in order to facilitate comparison.

One of the cargos of EVs is miRNAs. Multiple studies have indicated that there is a difference in the expression levels of exosomal miRNA, serum miRNA and could reflect the status of the releasing cell [128]. This lays the foundation for exosomal miRNA to act as a novel diagnostic biomarker for HCC [129]. MiRNA-21 is an oncogenic miRNA that is overexpressed in HCC. It is involved in the activation of the PTEN/Akt signaling pathway to increase the epithelial–mesenchymal transition, cell proliferation, and inhibition of apoptosis [130]. Wang et al. showed that the serum levels of exosome miRNA-21 were 2.21-fold higher in the HCC cohort compared with the chronic hepatitis B group, and 5.57-fold higher compared with the healthy population. High levels of miR-21 were also positively correlated with tumor stage [131]. MiRNA-638 is a tumor suppressor miRNA that is suppressed by tumor cells to activate metastatic cascade and to increase tumor survival [132]. In a randomized controlled study by Min Shi et al., the expression of exosomal miR-638 in the serum of HCC patients was reduced compared with healthy donors. In addition, they also found a negative association of serum exosomal miR-638 with tumor size, vascular infiltration, and TNM stage in HCC patients. Those with reduced serum exosomal miR-638 also had poor overall survival at 3 and 5 years compared with those with higher levels, supporting its role as a novel circulating diagnostic and prognostic biomarker [133]. Another study showed that the levels of peripheral blood MVs were significantly increased in HCC patients compared with liver cirrhosis, and were correlated to the tumor size, progression, and stage of the disease. The ROC for microvesicle discriminating patients with early TNM stage I and relatively early TNM stage II HCC from cirrhosis was found to be 0.83 (95% CI 0.74–0.93) and 0.94 (95% CI 0.88–1.00), respectively [123].

Emerging evidence has shown that the EVs released by HCC contain cargo that modulates the tumor microenvironment, facilitating the invasion, proliferation, and survival of tumor cells. This makes the characterization of EVs a promising method to better determine the tumor biology in a noninvasive manner. Their therapeutic potential is also being explored to directly target the delivery cargo to HCC [126,127,134,135]. EVs could, for example, be made with miRNA known to have functions that are capable of turning on or off oncogenes or oncogenetic pathways, which can then be directly delivered to HCC. This was done, for example, through the incorporation of miR-335–5p, which inhibited the proliferation and invasion of HCC cells [136]. Several studies explored the therapeutic potential and effective delivery methods of EVs in the treatment of hepatitis B and HCC [137]. Li and colleagues showed that the mechanism of intercellular communication by EVs can be utilized to transmit the antiviral effect of interferon alpha from non-parenchymal liver cells to infected parenchymal cells, conferring resistance to HBV replication [137]. Such strategies are only beginning to emerge as potential therapeutics for use in the treatment of HCC.

## 5. Conclusions

The early detection of HCC at a curative stage is of paramount importance. Unlike HCV infection, HBV infection currently has no effective curative therapies (HBsAg and HBV DNA loss), which leaves patients with chronic HBV at increased risk for the development of HCC. A further understanding of both the biology of the hepatitis B virus as well as the mechanisms that lead to HBV-HCC, are greatly needed in order to design better therapies for primary prevention [138]. Novel liquid biopsy methods to investigate in HCC include the detection of shed tumor components into the peripheral blood circulation, including CTC, cfDNA, and miRNAs, and exosomes with their cargo. The molecular profiling of CTC and EV cargo seems to have a promising role that will give further insight into tumor biology, which could then theoretically change its management. Further research should investigate better risk stratification models and the development of novel blood-based biomarkers, which could be useful for the early diagnosis, management, and post-treatment surveillance of HBV-associated HCC.

## 6. Patents

None.

## Figures and Tables

**Table 1:** Indications for surveillance in hepatitis B virus (HBV) patients per the American Association of the Study of Liver Disease (AASLD) guidelines [3,4].

Asian males over the age of 40
Asian females over the age of 50
African and/or North American blacks
HBV carrier with a family history of HCC in a first degree relative or concurrent hepatitis D infection
Hepatitis B with F3 fibrosis or cirrhosis

**Table 2:** Risk factors for development of HCC.

Host Factors
Older age
Male sex
Genetics/ethnicity
Diabetes/obesity
**Environmental Factors**
Smoking
Aflatoxin
Aristolochic acid
**Viral Factors**
HBV integration site into host DNA (*TERT*, *MLL4*, and *CCNE1*)
Virally translated proteins (HBsAg, HBx, HBeAg, and HBcAg)
HBV Genotype C

## Data Availability

Not applicable.

## Abbreviations

AASLD	American Association for the study of Liver Diseases
AFP	alpha fetoprotein
AFP-L1/L2/L3	isoforms of alpha fetoprotein
AFP-L3%	Fraction of AFP-L3 to total AFP
ALT	Alanine aminotransferase
AUC	Area under the curve
AUROC	Area under the receiver operating characteristic
Bcl-xL	B-cell lymphma-extra large protein
Bcl-2	B-cell lymphoma-2 protein
BMI	Body mass index
CAMD	Cirrhosis, age, male sex diabetes scoring system
cccDNA	covalently closed circular DNA
cfDNA	cell free DNA
ctDNA	circulating tumor DNA
CTC	Circulating tumor cells
CU-HCC	Chinese University Hepatocellular Carcinoma ScoreDDB1
DCP	des-gamma-carboxy prothrombin
EASL	European Association for the study of the Liver
ELISA	Enzyme-Linked Immunosorbent Assay
EpCAM	Epithelial cell adhesion molecule
EV	Extracellular Vesicle
GAG-HCC	Guide with age, gender, HBV DNA, core promoter mutations and cirrhosis score
GPC-73	Golgi Protein Complex 73
HBeAg	Hepatitis B e antigen
HBsAg	Hepatitis B surface antigen
HBx	Hepatitis B x protein
HBV	Hepatitis B virus
HBV-HCC	Hepatitis B related hepatocellular carcinoma
HCC	Hepatocellular carcinoma
KC	Kuppfer cell
miRNA	micro RNA
MISEV	Minimal information for studies of extracellular vesicles
NAFLD	Non-alcoholic fatty liver disease
NASH	Non-alcoholic steatohepatitis
OPN	Osteopontin
PAGE-B	Platelets, age, gender scoring system
PIVKA-II	Protein induced by vitamin K absence or antagonist-II
qRT-PCR	Quantitative reverse transcriptase polymerase chain reaction
REACH-B	Risk estimation for hepatocellular carcinoma in chronic hepatitis B
REVEAL-HBV	Risk evaluation of viral load elevation and associated liver disease/cancer in HBV
RISC	RNA-induced silencing complex
RNA	ribonucleic acid
RT-PCR	Reverse transcriptase polymerase chain reaction
SEC	Sinusoidral endothelial cells
TNM	TNM Classification of Malignant Tumors
